# In vitro membrane stabilizing, thrombolytic and antioxidant potentials of *Drynaria quercifolia* L., a remedial plant of the Garo tribal people of Bangladesh

**DOI:** 10.1186/s12906-016-1170-5

**Published:** 2016-07-04

**Authors:** Farjana Rahman Chaity, Mahbuba Khatun, Mohammad Sharifur Rahman

**Affiliations:** Phytochemical Research Laboratory, Department of Pharmaceutical Chemistry, Faculty of Pharmacy, University of Dhaka, Dhaka, 1000 Bangladesh; Department of Population Sciences, University of Dhaka, Dhaka, 1000 Bangladesh

**Keywords:** *Drynaria quercifolia*, Membrane stabilizing, Thrombolytic, DPPH, Hydrogen peroxide, ABTS, FRAP, Antioxidant, The Garo

## Abstract

**Background:**

*Drynaria quercifolia* L. (Family- Polypodiaceae) is a fern grows in Bangladesh used in traditional healing by the Garo tribe of Mymensingh district. In the current study, rhizomes and fertile foliage fronds of this plant have been investigated comprehensively to assess their *in vitro* membrane stabilizing, thrombolytic and antioxidant properties.

**Methods:**

Rhizomes and fertile foliage fronds of *D. quercifolia* were collected, dried, powdered and extracted with methanol. Later on, crude methanol extracts of the plant parts were fractionated into petroleum ether, carbon tetrachloride, chloroform and aqueous soluble fractions. The extractives were then subjected to membrane stabilizing, thrombolytic and antioxidant assays.

**Results:**

In membrane stabilizing assay, crude methanol extracts of rhizomes and fertile foliage fronds and their petroleum ether fractions were found to be very effective for stabilizing erythrocyte membrane in hypotonic solution. In case of thrombolytic study, crude methanol extract of rhizomes and its aqueous fraction exhibited noticeable clot lysis. However, in antioxidant assays, crude methanol extracts of the tested plant parts and their aqueous fractions exhibited potent 1,1-diphenyl-2-picrylhydrazyl (DPPH), hydrogen peroxide and 2, 2’-azinobis (3-ethylbenzothiazoline sulphonic acid) (ABTS) radical scavenging activity. Besides, these extractives also displayed substantial ferric reducing potential in ferric reducing antioxidant power (FRAP) assay. Crude methanol extracts of the plant parts and their aqueous fractions were also found rich in phenolics.

**Conclusion:**

This study demonstrates the medicinal potentials of *D. quercifolia* and justifies the local uses of it by the Garo tribal people of Bangladesh for multiple disease management.

## Background

Medicinal plants are always very promising for the development of new drugs. To distinguish any plant possessing medicinal quality, proper scientific screening is essential. Traditionally different plants are known to have different efficacy for treating various types of diseases. If the right plant is known for healing a particular disease, attempts should be made to isolate the bioactive lead molecule(s) from the plant [1].

Almost 30 tribal communities are living at different parts of Bangladesh. The Garo is one of them and considered as an ethnic group of ‘Tibbeti Borman’, belonging to the Mongolian human race. They are now living in Mymensingh, Tangail, Netrokona, Sylhet and Sunamgonj districts. They have their own curative practices involving many medicinal plants as evident from some ethnobotanical surveys [2, 3]. These plants can be systematically evaluated to explore their healing abilities. With this view, *Drynaria quercifolia* (L.) J. Sm., a medicinal plant used by the Garo tribe, was selected to perceive its medicinal properties through chemical and biological assays.

*D. quercifolia* (L.) J. Sm. (Bengali name: Bandor shoal, Pankhiraj) belongs to a large family of ferns, Polypodiaceae, having 56 genera and 1200 species distributed throughout the world. It is native to Bangladesh, India, Southeast Asia, Malaysia, Indonesia, Australia, the Philippines, New Guinea, etc. [4]. This epiphytic or lithophytic fern is available in wet tropical environments and generally grows on wall, tree or rock anchored by rhizome. It has two types of fronds known as fertile foliage fronds and sterile nest fronds. This plant is traditionally used to heal typhoid, hectic fever, cough, dyspepsia, jaundice, diabetes, tuberculosis, throat infection, etc. [5–8]. Besides, it has been reported to have antifertility, analgesic, antiedematous, antibacterial, etc. activities [6, 9–12]. Preliminary phytochemical screening has revealed the presence of alkaloids, glycosides, saponins, amino acids, flavonoids, triterpenes, phytosterols and carbohydrates [11, 13].

The aim of the present study is to investigate the rhizomes and the fertile foliage fronds of *D. quercifolia* for their membrane stabilizing, thrombolytic and antioxidant activities comprehensively. This might explore the remedial potential of this plant to a great extent.

## Methods

### Collection and identification of plant materials

*D. quercifolia* is abundant in the Garo Hill of Mymensingh, Bangladesh and was collected from there on June, 2013. It was officially identified by an expert taxonomist at Bangladesh National Herbarium (BNH), Mirpur Road-1, Dhaka (http://www.bnh.gov.bd), which is a government institute for providing plant identification service nationally. An accession number DACB -38386 was provided by BNH after identification of the submitted voucher specimen of the plant. The specimen has been preserved there for future references.

### Extraction

**Fig. 1:**
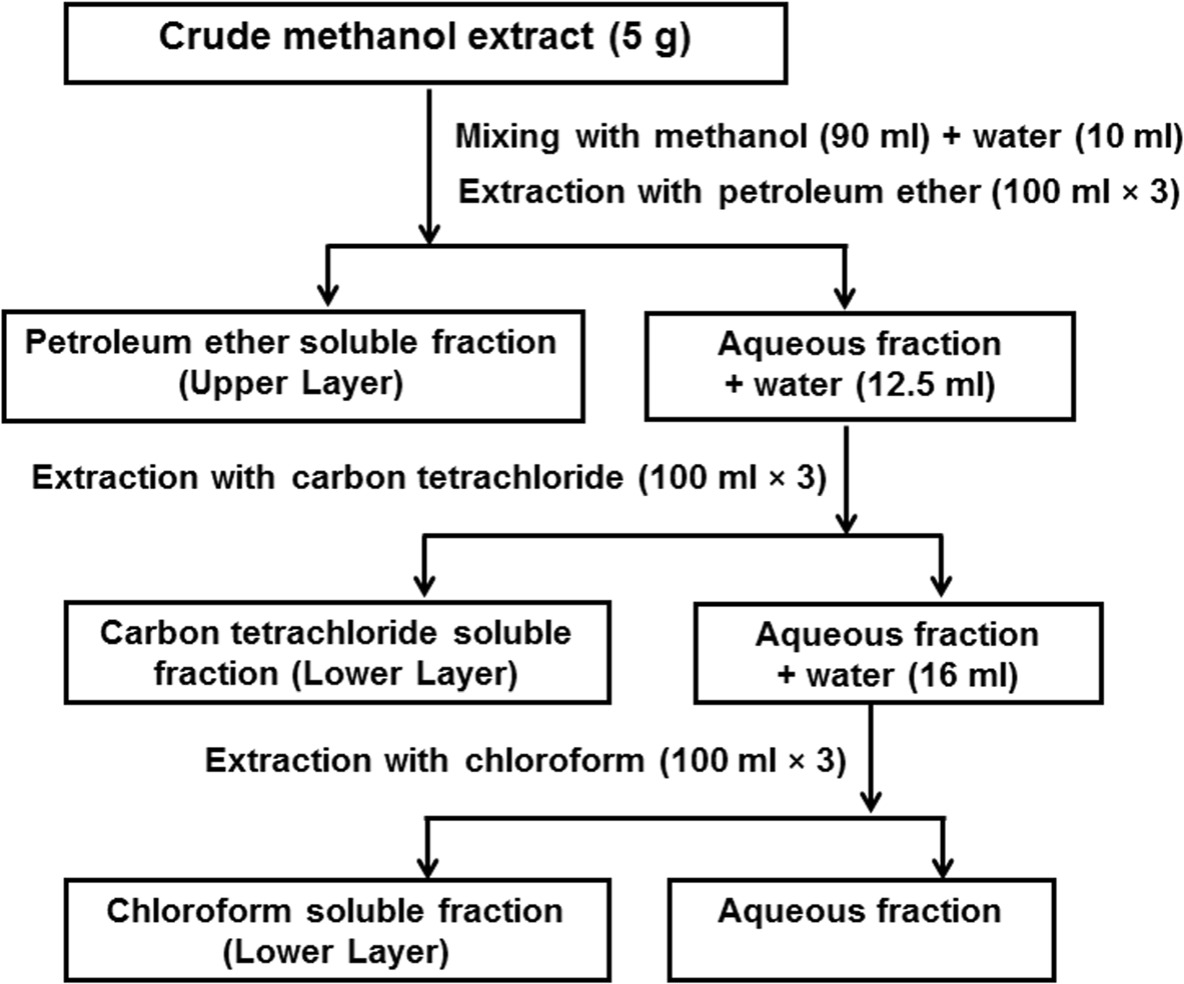
Fractionation. Schematic representation of the modified Kupchan partitioning of crude methanol extracts

The collected rhizomes and fertile foliage fronds were chopped, dried and powdered separately. 500 g of the each powdered materials were soaked separately in 1.5 L of methanol at room temperature for 7 days. The extracts were filtered through cotton plug and concentrated with a rotary evaporator. An aliquot (5 g) of the concentrated methanol extract was fractionated by the modified Kupchan method [14] into petroleum ether, carbon tetrachloride, chloroform and aqueous soluble fractions (Fig. 1) followed by solvent evaporation.

### Membrane stabilizing activity

This method was developed by Shinde et al. to investigate the membrane stabilizing activity of plant extractives using erythrocytes [15]. Briefly, 5 ml of the whole blood was collected from healthy human volunteers in a tube containing dipotassium salt of EDTA (2.2 mg/ml of blood). The blood was centrifuged to collect blood cells and then washed three times with isotonic solution (154 mM NaCl) in 10 mM sodium phosphate buffer (pH 7.4) through centrifugation (10 min at 3000 g) using the same volume as supernatant. Finally, it was resuspended in the equal volume of this isotonic buffer solution. 0.5 ml of the suspension was mixed with 5 ml of hypotonic solution (50 mM NaCl) in 10 mM sodium phosphate buffered saline (pH 7.4) containing either the extract (2 mg/ml) or reference drug, acetylsalicylic acid (0.1 mg/ml). The control sample had 0.5 ml of erythrocytes mixed with hypotonic-buffered saline alone. The mixture was incubated for 10 min at room temperature, centrifuged for 10 min at 3000 g and the optical density (OD) of the supernatant was measured at 540 nm by using a UV-visible spectrophotometer. The percentage inhibition of hemolysis was calculated using the following equation

$$ \%\ \mathrm{inhibition}\ \mathrm{of}\ \mathrm{hemolysis} = \left\{\left({\mathrm{OD}}_{\mathrm{control}}\hbox{-}\ {\mathrm{OD}}_{\mathrm{test}\ \mathrm{samples}}\right)/{\mathrm{OD}}_{\mathrm{control}}\right\} \times 100 $$

### Thrombolytic activity

The extractives were evaluated for thrombolytic activity using human blood by a method described earlier by Prasad et al. [16]. For this assay, 5 ml of venous blood was collected from healthy volunteers and dispersed in sterile pre-weighed microcentrifuge tubes (0.5 ml/tube) followed by incubation at 37 °C for 45 min. After clot formation, the serum was removed carefully and tubes were weighed. 100 μl of each of the extractives (2 mg/100 μl of water) was added in each tube. Here, 100 μl of streptokinase (equivalent to 30,000 I.U.) (Altepase®, Beacon Pharmaceuticals Ltd., Bangladesh) and 100 μl of distilled water were used as positive control and negative control, respectively. After incubation of the tubes at 37 °C for 90 min, the developed fluid from the clot was removed and tubes were again weighed to measure the clot lysis. Percentage of clot lysis was expressed as:

$$ \%\ \mathrm{thrombolysis} = \left(\mathrm{weight}\ \mathrm{of}\ \mathrm{clot}\ \mathrm{after}\ \mathrm{treatment}\ /\mathrm{weight}\ \mathrm{of}\ \mathrm{clot}\ \mathrm{before}\ \mathrm{treatment}\right) \times 100 $$

### 1,1-Diphenyl-2-picrylhydrazyl (DPPH) free radical scavenging activity

The stable DPPH was used for the determination of free radical scavenging activity [17]. In this assay, a methanol solution of the extract or standard (2 ml) at different concentrations (500 to 15.625 μg/ml) was mixed with a 3 ml of DPPH solution (20 μg/ml methanol). Absorbance was determined at 517 nm by using a UV-visible spectrophotometer after 20 min keeping in dark. The percent inhibition was calculated from [(Ao–A_1_)/Ao] × 100, where Ao is the absorbance of the control and A_1_ is the absorbance of the test sample. Here, ascorbic acid was used as standard. IC_50_ (half maximal inhibitory concentration) was calculated from the graph plotted inhibition percentage against extract concentration.

The antioxidant activity index (AAI) was calculated from the following equation [18]:

$$ \mathrm{A}\mathrm{A}\mathrm{I} = \mathrm{Final}\ \mathrm{concentration}\ \mathrm{of}\ \mathrm{DPPH}\ \left(\upmu \mathrm{g}/\mathrm{ml}\right)/{\mathrm{IC}}_{50}\left(\upmu \mathrm{g}/\mathrm{ml}\right) $$

### Hydrogen peroxide free radical scavenging activity

The free radical scavenging activity of the plant extractives was also examined by using hydrogen peroxide free radical [19]. In this experiment, 43 mM hydrogen peroxide solution was prepared in phosphate buffer saline (pH 7.4). Ascorbic acid (standard) and the extract solution were arranged separately at concentration from 500 to 15.625 μg/ml. An aliquot of either standard or extract solutions (3.4 ml) was mixed with 0.6 ml of hydrogen peroxide solution. The reaction mixtures were incubated at room temperature for 10 min and the absorbance was determined at 230 nm by using a UV-visible spectrophotometer. The percentage inhibitory activity (I %) was calculated from [(Ao–A_1_)/Ao] × 100, where Ao is the absorbance of the control and A_1_ is the absorbance of the test sample. Here, ascorbic acid was used as standard. IC_50_ was calculated from the graph plotted inhibition percentage against extract concentration.

### 2, 2’-Azinobis (3-ethylbenzothiazoline sulphonic acid) (ABTS) radical scavenging activity

ABTS scavenging activity was determined by using a method described by Re et al. [20] with minor modification. The stock solution was comprised of 7 mM ABTS solution and 2.45 mM potassium persulfate (K_2_S_2_O_8_). The working solution was kept at room temperature in the dark for 16 h. The ABTS solution was adjusted with distilled water to obtain an absorbance of 0.70 ± 0.02 at 734 nm. Fresh ABTS solution was prepared for each assay. During test, 0.1 ml of extract or standard of different concentrations (500-15.625 μg/ml) was allowed to react with 2.9 ml of the ABTS solution in the dark at room temperature for 7 min. Then the absorbance was measured at 734 nm with the help of a UV-visible spectrophotometer. The percent inhibition was calculated from [(Ao–A_1_)/Ao] × 100, where Ao is the absorbance of the control and A_1_ is the absorbance of the test sample. Ascorbic acid was used as standard.

### Ferric reducing antioxidant power (FRAP) assay

The ferric reducing activity was conducted by a modified method of Benzie and Strain [21]. The stock solution was comprised of 300 mM acetate buffer (3.1 g C_2_H_3_NaO_2_ · 3H_2_O and 16 ml C_2_H_4_O_2_), pH 3.6, 10 mM 2,4,6-tripyridyl-s-triazine (TPTZ) solution in 40 mM HCl and 20 mM FeCl_3_ · 6H_2_O solution. The freshly prepared working solution was a mixture of 25 ml acetate buffer, 2.5 ml TPTZ and 2.5 ml FeCl_3_ · 6H_2_O. The temperature of the solution was maintained at 37 °C before use. 200 μl of extract (2.5 mg/ml) or ascorbic acid (100 μg/ml) was allowed to react with 2800 μl of the FRAP solution for 30 min in the dark condition. The absorbance of the colored product (ferrous tripyridyltriazine complex) was taken at 593 nm by using a UV-visible spectrophotometer. A standard curve was prepared for FeSO_4_ (0-1000 μM) and the results were expressed in μmol of Fe^+2^/g dry mass.

### Total phenolic content determination

Total phenolic content of the extractives was measured by using Folin-Ciocalteu reagent as an oxidizing agent and gallic acid as a standard [22]. In this assay, 0.5 ml of plant extract (2 mg/ml) in water was mixed with 2.5 ml of Folin-Ciocalteu reagent (10 times diluted with water) and 2 ml of sodium carbonate (7.5 % w/v) solution. After 20 min of incubation at room temperature, the absorbance was measured at 760 nm by using a UV-visible spectrophotometer. Total phenolic content was determined by calibration curve obtained from measuring the known concentrations of gallic acid (0-100 μg/ml) and expressed as mg of GAE (gallic acid equivalent)/g of the dried extract.

### Statistical analysis

Three replicates of each sample were used for statistical analysis and the values are reported as mean ± standard deviation (SD).

## Results

### Membrane stabilizing activity

**Fig. 2:**
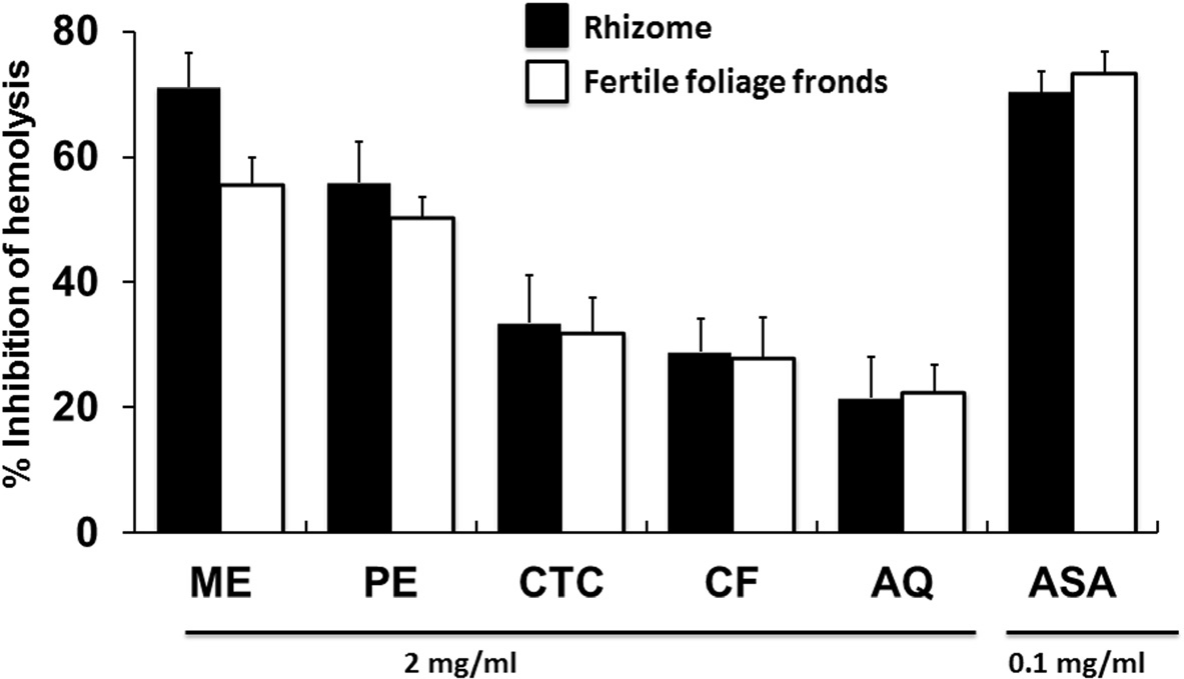
Membrane stabilizing activity of *D. quercifolia* and the standard on hypotonic solution-induced hemolysis of erythrocyte membrane. Here, ME: crude methanol extract; PE- petroleum ether soluble fraction; CTC- carbon tetrachloride soluble fraction; CF- chloroform soluble fraction; AQ-aqueous fraction; ASA- acetylsalicylic acid (standard)

The highest level of membrane stabilizing activity was exhibited by the crude methanol extract of rhizomes (70.9 %). The moderate membrane protecting activity (considering >50 % inhibition of hemolysis) was noticed by the crude methanol extract of fertile foliage fronds and petroleum ether fractions of both the tested parts of the plant as shown in Fig. 2.

### Thrombolytic activity

**Fig. 3:**
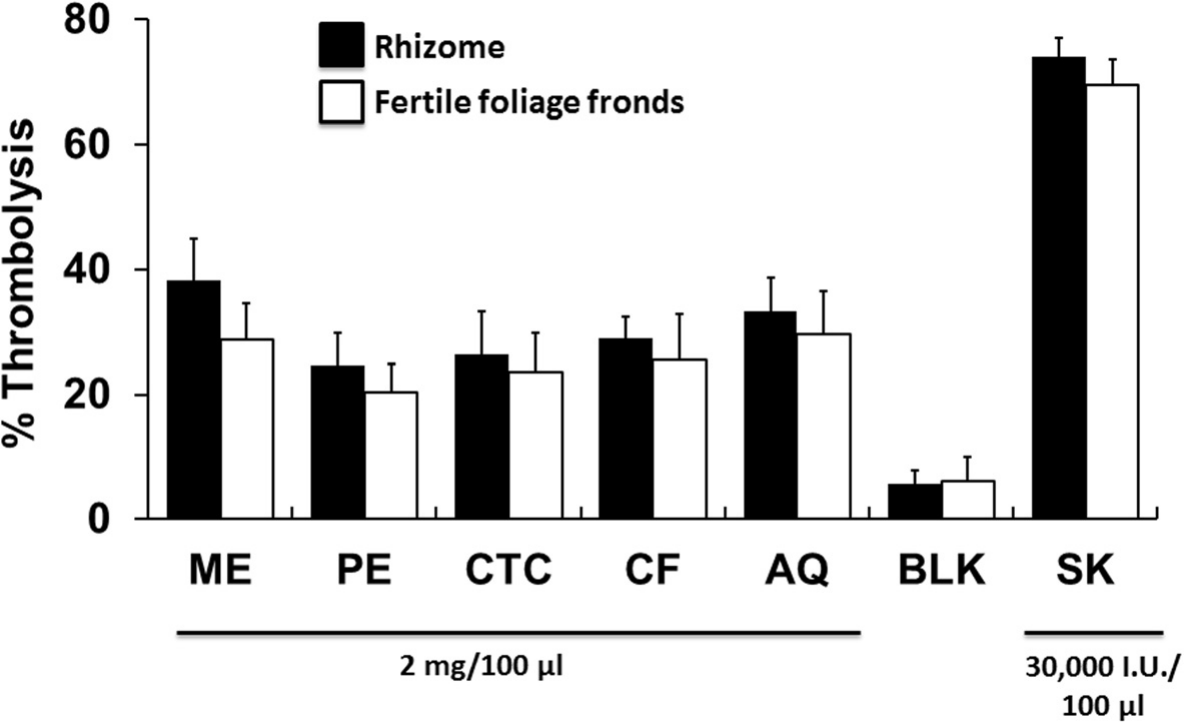
Thrombolytic activity of *D. quercifolia* and the standard. Here, ME: crude methanol extract; PE- petroleum ether soluble fraction; CTC- carbon tetrachloride soluble fraction; CF- chloroform soluble fraction; AQ-aqueous fraction; BLK-Blank; SK-streptokinase (standard)

The thrombolytic activity of the plant extractives is shown in Fig. 3. Prominent thrombolysis was demonstrated by the crude methanol extract of rhizomes (38.34 %) and its aqueous fraction (33.32 %). Moderate clot lysis ability (considering >25 % lysis) was seen for the crude methanol extract and aqueous fraction of the fertile foliage fronds. Chloroform fractions of both parts of the fern were also moderate in thrombolysis.

### DPPH free radical scavenging activity

**Fig. 4:**
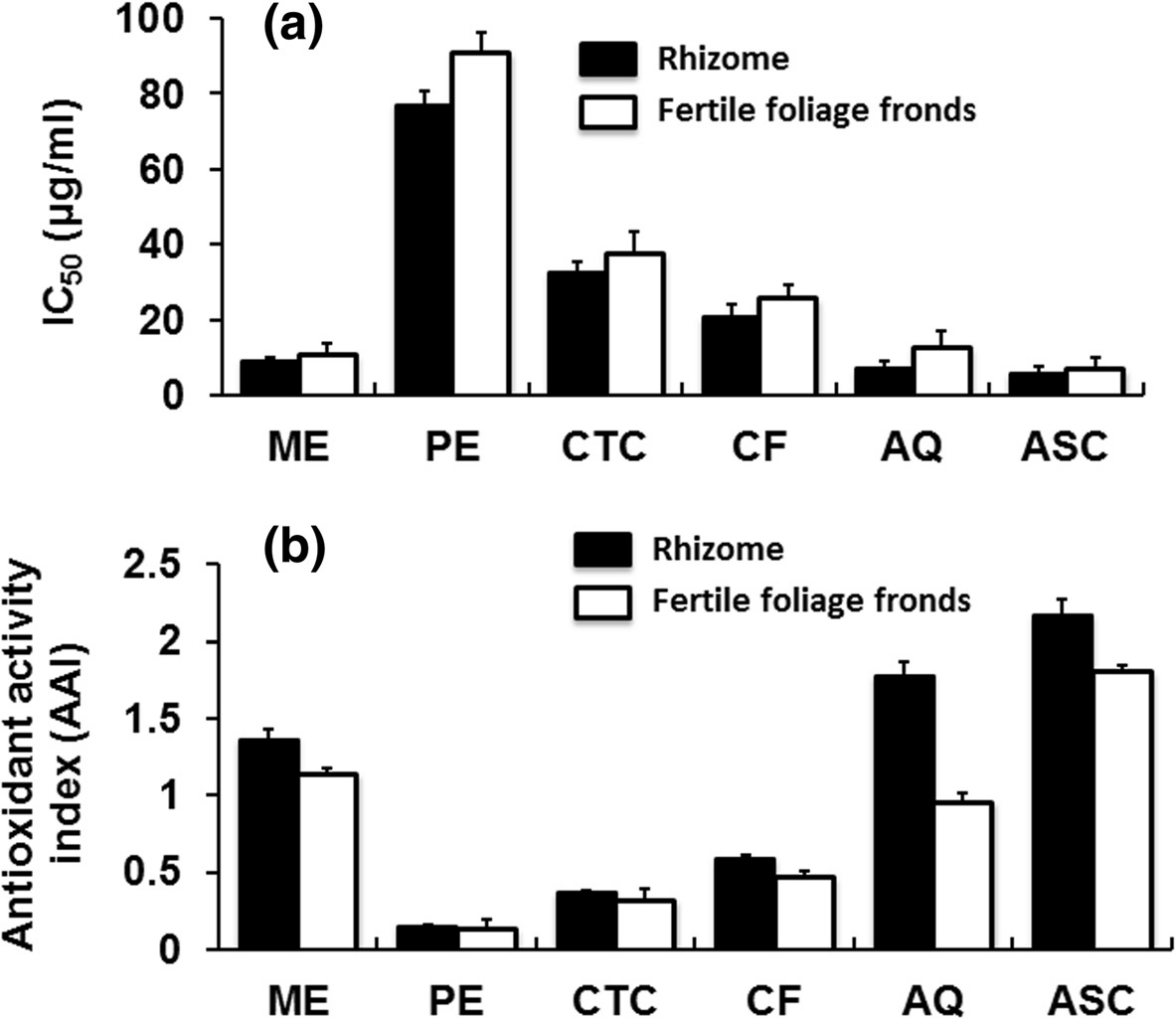
DPPH free radical scavenging assay of *D. quercifolia* and the standard representing IC_50_ values (**a**) and antioxidant activity index (**b**). Here, ME: crude methanol extract; PE- petroleum ether soluble fraction; CTC- carbon tetrachloride soluble fraction; CF- chloroform soluble fraction; AQ-aqueous fraction; ASC-ascorbic acid (standard)

The free radical scavenging activities are illustrated in Fig. 4a. Crude methanol extracts of both parts of the plant and their aqueous fractions exhibited noticeable free radical scavenging activity with the IC_50_ values less than 15 μg/ml, respectively. The antioxidant activity index (AAI) was also calculated using both concentration of DPPH and IC_50_ to determine a constant for each antioxidant and shown in the Fig. 4b. Among the tested extractives, the aqueous fraction of the rhizome displayed the highest AAI (1.77), whereas crude methanol extracts of both parts of the fern and aqueous fraction of the fertile foliage fronds demonstrated moderate antioxidant activity having AAI > 0.9.

### Hydrogen peroxide free radical scavenging activity

**Fig. 5:**
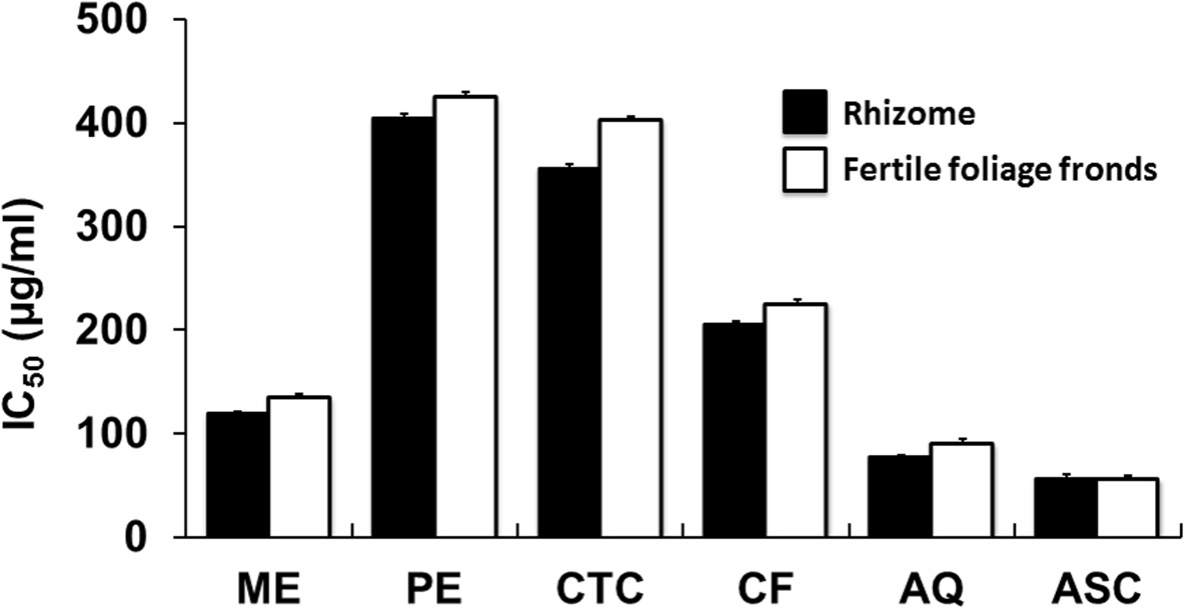
Hydrogen peroxide free radical scavenging assay of *D. quercifolia* and the standard. Here, ME: crude methanol extract; PE- petroleum ether soluble fraction; CTC- carbon tetrachloride soluble fraction; CF- chloroform soluble fraction; AQ-aqueous fraction; ASC-ascorbic acid (standard)

The hydrogen peroxide free radical scavenging activities are shown in Fig. 5. Aqueous fractions of both rhizomes and fertile foliage fronds showed noticeable radical scavenging activity with IC_50_ values 77.36 ± 2.2 and 90.33 ± 4.1 μg/ml, respectively. Besides, crude methanol extracts displayed moderate free radical scavenging activity having IC_50_ values less than 150 μg/ml.

### ABTS radical scavenging activity

**Fig. 6:**
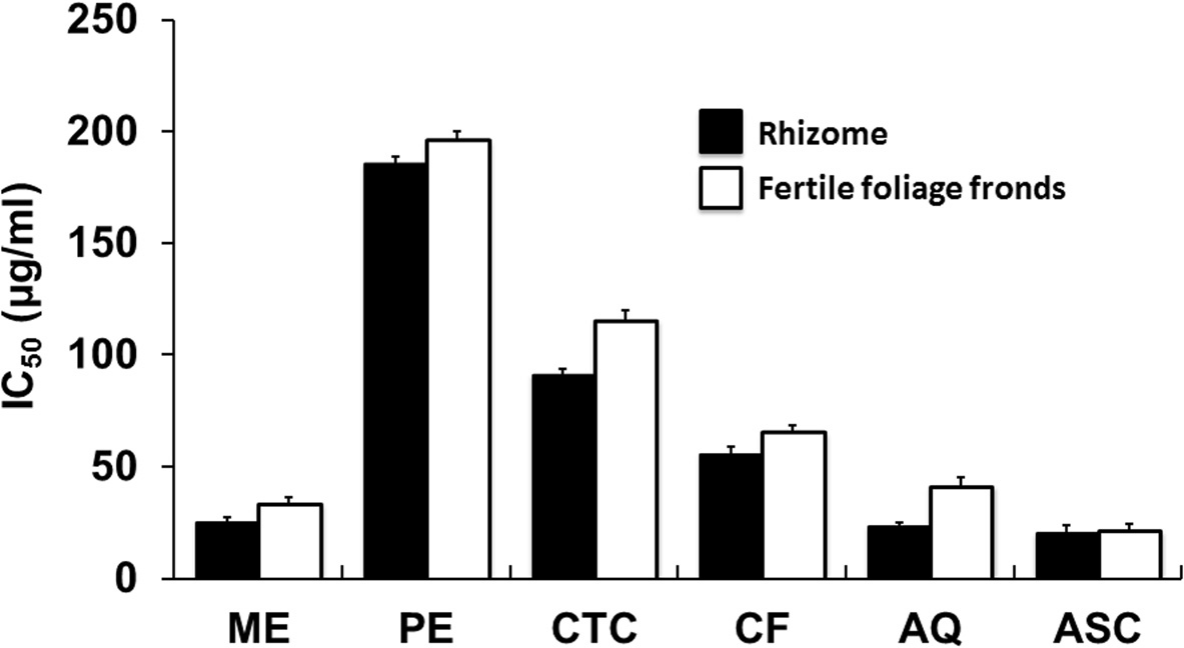
ABTS free radical scavenging assay of *D. quercifolia* and the standard. Here, ME: crude methanol extract; PE- petroleum ether soluble fraction; CTC- carbon tetrachloride soluble fraction; CF- chloroform soluble fraction; AQ-aqueous fraction; ASC-ascorbic acid (standard)

The ABTS radical scavenging activities of the tested samples are presented in Fig. 6. Aqueous fraction and crude methanol extract of rhizomes were effective scavengers of the ABTS radical displaying the IC_50_ values 23.29 ± 1.85 and 25.4 ± 2.25 μg/ml, respectively.

### Ferric reducing antioxidant power (FRAP) assay

In the present study, the equation generated for the standard curve of FeSO_4_ solution is as follows:

$$ \mathrm{y}=0.001\mathrm{x}{\textstyle -}0.0007,{\mathrm{R}}^2=0.9987 $$

**Fig. 7:**
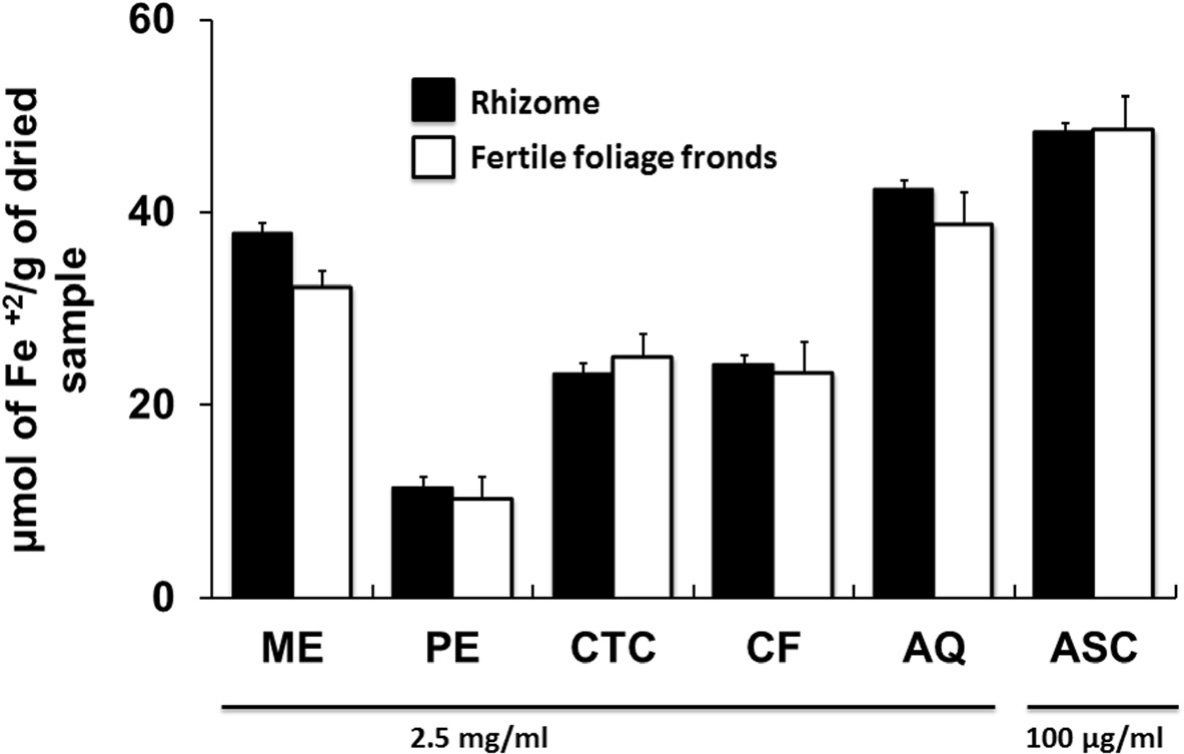
FRAP assay of *D. quercifolia* and the standard. Here, ME: crude methanol extract; PE- petroleum ether soluble fraction; CTC- carbon tetrachloride soluble fraction; CF- chloroform soluble fraction; AQ-aqueous fraction; ASC-ascorbic acid (standard)

Where x is the absorbance and y is the FeSO_4_ equivalent μmol of Fe^+2^/g of dried extract. Total ferric reducing power of the extractives is shown in Fig. 7. The highest reducing ability was seen for the aqueous fraction and crude methanol extract of rhizomes having FRAP value 42.36 ± 1.36 and 37.88 ± 1.22 μmol of Fe^+2^/g of dried extract, respectively.

### Total phenolic content determination

The standard curve of gallic acid provided the equation:

$$ \mathrm{y} = 0.008\mathrm{x} + 0.0071,\ {\mathrm{R}}^2 = 0.9986 $$

**Fig. 8:**
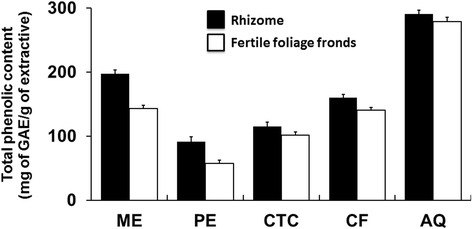
Total phenolic content of *D. quercifolia*. Here, ME: crude methanol extract; PE- petroleum ether soluble fraction; CTC- carbon tetrachloride soluble fraction; CF- chloroform soluble fraction; AQ-aqueous fraction; ASA- acetylsalicylic acid (standard); GAE- gallic acid equivalent

Where x is the absorbance and y is the gallic acid equivalent (mg/g). The phenolic content level of the extractives is shown in Fig. 8. The highest level of phenolic content was determined in aqueous fractions. Crude methanol extracts were also found rich in phenolics.

## Discussion

The Garo is a tribal community of Bangladesh. Their family pattern, norms, foods, festivals, etc. are different from other tribal communities and mainstream Bangladeshi common people [3]. The healers of this tribe use many medicinal plants for treating their people. Recently some ethnobotanical surveys have been conducted on this [2, 6], which have revealed the studied fern, *D. quercifolia*, as one of the medicinally important plants of the Garo tribe.

Inflammation is a very complex biological state. Chronic inflammation may be associated with aging, cancer, adipogenesis, diabetes, cardiovascular problems, lung disease, etc. [23–25]. Membrane stabilization assay of erythrocytes is a very popular tool to investigate the anitiinflammatory potential of the plant extract. When erythrocytes are exposed to hypotonic medium, heat, methyl salicylate, phenylhydrazine, etc., the lysis of their membrane occurs. It results in the leakage of serum protein and fluids into the tissues instigating inflammation [26]. So, the compounds capable of membrane stabilization might be very suitable as antiinflammatory agents [15]. Crude methanol extract of rhizomes demonstrated noticeable membrane stabilizing property (Fig. 2). Moderate membrane protecting activity was displayed by the crude methanol extract of fertile foliage fronds and petroleum ether fractions of both parts of the fern. The antiinflammatory potential of this plant was also partially verified earlier by another preliminary study using only ethanol extract of rhizomes on mice model [9]. However, active inflammation of the gastroduodenal mucosa is a cause of dyspepsia [27]. *D. quercifolia* is well recognized to treat dyspepsia [7], which might be linked with antiinflammatory potential of this fern. Several flavonoids and triterpenes have been reported earlier to have antiinflammatory activity [28, 29]. As flavonoids and triterpenes are also present in *D. quercifolia* [13, 30], it might be a reason for its membrane stabilizing antiinflammatory potential.

Thrombus development leads to many vascular complexities including myocardial infarction, stroke, deep vein thrombosis, renal vein thrombosis, portal vein thrombosis, etc. which might result in death. Tissue plasminogen activator, urokinase, streptokinase, etc. are used currently for treating thrombosis but better thrombolytic agents are still a demand of time [31, 32]. In the current study, higher thrombolytic activity was exhibited by crude methanol extracts and aqueous fractions of both rhizomes and fertile foliage fronds (Fig. 3). This plant was previously reported to have significant hypolipidemic activity on diabetic rats [33]. The thrombolytic activity of the plant along with lipid lowering ability might contribute to maintain a healthy cardiovascular system [34]. It has been noticed earlier that flavonoids might have significant potentials of displaying thrombolytic activity [35]. The abundance of flavonoids in *D. quercifolia* might be a reason for its thrombolytic ability [13].

Free radicals become notorious when produced in excess in vivo and cause oxidative damages. They can destroy immunity system and develop a wide range of diseases such as alzheimer's disease, parkinson's disease, complication in diabetes, rheumatoid arthritis, neuro-degeneration, cardiovascular complications, DNA damage, carcinogenesis, metabolic disorders, aging, etc. [36, 37]. To counteract the complications generated by free radicals, antioxidants might be very beneficial. Different fractions of *D. quercifolia* were subjected to DPPH and hydrogen peroxide free radical scavenging assays, where crude methanol extracts and their aqueous fractions scavenged the free radicals evidently (Figs. 4 and 5). On the other hand, the decolorization in ABTS assay reveals the capacity of an antioxidant species to donate electrons or hydrogen atoms to deactivate the radical species of ABTS [20]. Aqueous fractions and crude methanol extracts again displayed noticeable ABTS radical scavenging capacity (Fig. 6). However, FRAP assay measures the reducing potential, where a compound exerts its effect by donating hydrogen atom to ferric tripyridyltriazine complex and interferes the radical chain reaction [21]. In the present study, aqueous fractions and crude methanol extracts were very dominant for exhibiting ferric reducing potentials (Fig. 7).

It is well-known that phenolic compounds are very useful to serve as antioxidants [38]. So, the total phenolic content of extractives was assessed (Fig. 8). Higher level of phenolic content was seen in the aqueous fractions and crude methanol extracts of the plant. This finding might explain a reason for stronger antioxidant potential of the high polar extractives.

Jaundice might cause accelerated generation of hydroxyl radicals in plasma and liver tissues as evident from an earlier study performed on common bile duct ligated jaundice rats [39]. *D. quercifolia* is traditionally used for curing jaundice [5] which might be related with the antioxidant potential of this fern. A higher level of free radicals and oxidative stress has a vital role in the deterioration of diabetic complications. Therefore, use of antioxidants reduces oxidative stress and helps in diabetes management [40]. The Garo healers use *D. quercifolia* to treat diabetes mellitus [6], which is very much reasonable in the context of antioxidant potential of this fern. Antioxidant treatment was reported very promising for quick alleviation of respiratory infection and inflammation [41]. Rhizomes and fronds of *D. quercifolia* are also locally used for treating tuberculosis and throat infections [8], which might be assisted with the antioxidant potential of this plant as evident from the present study along with its antibacterial potential as reported earlier [13].

## Conclusion

*D. quercifolia* is a medicinally important plant to the Garo tribal people of Bangladesh. In the present study, rhizomes and fertile foliage fronds of this fern have been studied comprehensively. Crude methanol extracts and petroleum ether fractions of rhizomes and fertile foliage fronds were very promising to show membrane stabilizing antiinflammatory potential. Besides, crude methanol extracts and aqueous fractions were very active for displaying thrombolytic activity. In addition, aqueous fractions and crude methanol extracts of both parts of the plant were very capable to scavenge the free radicals and reduce oxidized materials, which might be attributed to the high level of phenolic contents of the polar extractives. The outcomes of the present study add some values to the reported healing traits of this fern. Overall, the medicinal potential of this plant can be considered very substantial. Further comprehensive investigations are required to isolate bioactive compound(s) and know the underlying mechanisms. Herbal drug developers might also use these information for appropriate safety and efficacy trial in future.
